# Protein kinase B and extracellular signal-regulated kinase contribute to the chondroprotective effect of morroniside on osteoarthritis chondrocytes

**DOI:** 10.1111/jcmm.12559

**Published:** 2015-03-05

**Authors:** Liang Cheng, Guoqing Zeng, Zejun Liu, Bing Zhang, Xu Cui, Honghai Zhao, Xinpeng Zheng, Gang Song, Jian Kang, Chun Xia

**Affiliations:** a Zhongshan Hospital, University of XiamenXiamen, Fujian, China; b Taiping People’s Hospital of Dongguan, University of JinanDongguan, Guangdong, China; c School of Medicine, University of XiamenXiamen, Fujian, China

**Keywords:** morroniside, chondroprotective effect, AKT, ERK, human OA chondrocytes, rat OA model

## Abstract

Despite extensive studies on the multifaceted roles of morroniside, the main active constituent of iridoid glycoside from Corni Fructus, the effect of morroniside on osteoarthritis (OA) chondrocytes remains poorly understood. Here, we investigated the influence of morroniside on cultured human OA chondrocytes and a rat experimental model of OA. The results showed that morroniside enhanced the cell viability and the levels of proliferating cell nuclear antigen expression (PCNA), type II collagen and aggrecan in human OA chondrocytes, indicating that morroniside promoted chondrocyte survival and matrix synthesis. Furthermore, different doses of morroniside activated protein kinase B (AKT) and extracellular signal-regulated kinase (ERK) in human OA chondrocytes, and in turn, triggered AKT/S6 and ERK/P70S6K/S6 pathway, respectively. The PI3K/AKT inhibitor LY294002 or the MEK/ERK inhibitor U0126 attenuated the effect of morroniside on human OA chondrocytes, indicating that the activation of AKT and ERK contributed to the regulation of morroniside in human OA chondrocytes. In addition, the intra-articular injection of morroniside elevated the level of proteoglycans in cartilage matrix and the thickness of articular cartilage in a rat experimental model of OA, with the increase of AKT and ERK activation. As a consequence, morroniside has chondroprotective effect on OA chondrocytes, and may have the therapeutic potential for OA treatment.

## Introduction

Osteoarthritis (OA) is the most common degenerative disease of human articular cartilage. Chondrocyte death and the loss of extracellular matrix are the central features in articular cartilage degeneration during OA pathogenesis 1,2. The blockade of chondrocyte death and extracellular matrix depletion has become potential therapeutic targets for the prevention of OA 3,4.

**Figure 1:**
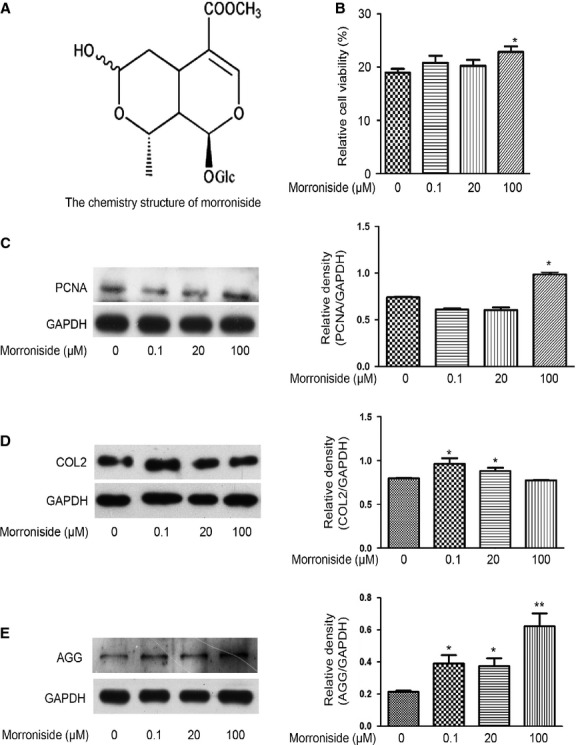
Morroniside promotes cell survival and matrix formation in human OA chondrocytes. Cells were treated with different dose of morroniside (0.1, 20 and 100 μM) for 24 hrs. (A) The chemistry structure of morroniside. (B) The cell viability was measures by the MTT assay. (C) PCNA expression was detected by western blotting analysis. (D and E) COL2 and AGG levels were detected by Western blotting analysis. The values represent the mean ± SEM of three to five independent experiments, each yielding similar results (*P < 0.05, **P < 0.01).

Morroniside is the main active constituent of iridoid glycoside from Corni Fructus (Fig.1A), and exhibits several bioactivities such as antioxidant and anti-inflammatory effects 5,6. Recently, it has been shown that morroniside can also protect cell against apoptosis and promote cell proliferation. Morroniside has reversed the apoptotic effect of H_2_O_2_ on human embryonic lung fibroblast cell growth 7. Morroniside has prevented peroxide-induced apoptosis of SH-SY5Y cells (a neuroblastoma cell line) 8. These results show that morroniside is involved in the regulation of apoptosis and protects some cell lines against apoptosis from harmful factors. However, the effect and regulatory mechanism of morroniside on chondrocytes, specifically, OA chondrocytes, remain understood.

The protein kinase B (AKT), which can be activated in response to growth factors and various extracellular stimuli and regulate many cellular processes including apoptosis, cell survival and angiogenesis, plays an important role in the metabolism of articular chondrocyte 9–13. The activation of AKT is more increased in normal than in OA chondrocytes and was required for both basal and insulin-induced type II collagen (COL2) expression in chondrocytes 11. Moreover, the expression of the AKT inhibitor called TRB3 (tribbles homolog, a pseudokinase that contains a truncated kinase domain and binds to AKT to inhibit AKT phosphorylation and activation) increases in human OA cartilage 12. Thus, it has been confirmed that the activation of AKT, at least in part, contributes to OA pathogenesis.

Additionally, activated extracellular signal-regulated kinase (ERK) is also involved in OA pathogenensis 14–16. For example, the MEK/ERK pathway has enhanced the production of a variety of pro-inflammatory cytokines in arthritic diseases by inducing and sustaining cartilage damage by negatively influencing the balance between cartilage destruction and cartilage repair 14. The inhibition of MEK in a rabbit experimental model of OA is associated with a reduction in the development of structural changes and in the levels of phospho-ERK1/2 (p-ERK1/2) in OA chondrocytes 15.

In the present study, we investigated the effect of morroniside on OA chondrocytes and the role of AKT and ERK signalling in the regulatory mechanism of morroniside in cultured human OA chondrocytes and a rat experimental model of OA. Our findings indicate that morroniside can protect OA chondrocytes against apoptosis and matrix depletion by the activation of AKT and ERK signalling, and may be beneficial to understanding the effect and regulatory mechanism of morroniside on OA chondrocytes,.

## Materials and methods

### Reagents and antibodies

Inhibitors and antibodies against AKT, p-AKT-Ser473, GAPDH, ERK1/2, p-ERK1/2(thr202/tyr204), p-mammalian target of rapamycin (mTOR; ser2448), p-P70S6K(Thr389) and p-S6 (Ser235/236) were obtained from Cell Signaling Technology (Beverly, MA, USA). Antibodies against proliferating cell nuclear antigen (PCNA), aggrecan (AGG) and COL2 were obtained from Santa Cruz Biotechnology (Santa Cruz, CA, USA). Collagenase II and DMEM/F12 were obtained from Gibco (Carlsbad, CA, USA). Morroniside was purchased from Chengdu Best Reagent Co., Ltd. (Chengdu, China) and prepared as a 100 mM stock solution in water. The required doses of morroniside for individual experiments were made by further dilution of the 100 mM stock solution with culture medium as needed. Unless otherwise specified, the rest of the reagents were purchased from Sigma-Aldrich (St. Louis, MO, USA).

### Human OA chondrocyte isolation and culture

Ethical approval for the study was obtained from the Ethics Committee of Zhongshan Hospital, Xiamen University (ID no. 20100426), China. After receiving all patient consent and in accordance with the hospital ethical guidelines, human OA cartilage was obtained from 26 patients (aged 60–72 years, 6 males and 20 females) with advanced OA who were undergoing total knee replacement surgery (Table1). The OA patients were diagnosed based on the criteria developed by the American College of Rheumatology Diagnostic Subcommittee for OA, and had not taken non-steroidal anti-inflammatory drugs or steroids for at least 2 weeks prior to surgery or had any intra-articular injection for at least 1 month prior to surgery. Articular cartilage was dissected from the femoral condyle and tibial plateau (Fig. S1A), and was stored in liquid nitrogen for chondrocyte culture. As described previously 17,18, primary chondrocytes were cultured to 80% confluence and plated in 60-mm Petri dishes or 96-well plates. All of our clinical studies have been conducted according to the principles expressed in the Declaration of Helsinki.

**Table 1:** Information of OA patients with total knee replacement surgery

Age (year)	Case	Gender		Duration of OA (year)		K.L. Image criterion*		Pro-treatment arthroscopy
		M	F	≤3	>3	III	IV	
60−	10	2	8	3	7	2	8	3
65−	16	4	12	5	11	3	13	2

* K.L. Image criterion: Kellgren and Lawrecne criterion.

### Cell viability analysis

Cells were plated in 96-well plates (3 × 10^3^ cells/well) and starved with medium without serum for 6 hrs. The medium was then replaced with fresh culture medium and the cells were treated with different doses of morroniside in the presence or absence of inhibitors for a further 24 hrs, followed by the 3-(4,5-dimethylthiazol-2-y1)-2,5-diphenyltetrazolium bromide (MTT) assay, which is a method to measure cytotoxicity, proliferation and activation 19. The end product of the MTT assay was dissolved in dimethylformamide and quantified spectrophotometrically at a wavelength of 490 nm with a reference wavelength of 630 nm. The OD values correspond to the number of viable cells 19,20.

### Protein extraction and western blotting analysis

Cells collected by centrifugation were lysed as previously described 13. Protein extracts were subjected to SDS-PAGE (8–10%) and transferred to a nitrocellulose membrane for western blotting analysis 13. The signal was monitored using a chemiluminescent detection system according to the manufacturer’s instructions (Pierce, Rockford, IL, USA).

### Establishment of a rat experimental model of OA

This study was carried out in strict accordance with the recommendations in the Guide for the Care and Use of Laboratory Animals of the National Institutes of Health. The protocol was approved by the Committee on the Ethics of Animal Experiments of the University of Xiamen (ID no. 20110920). 9-week-old male Sprague–Dawley rats (250–300 g) were used in the following experiments. The animals were acclimatized to the laboratory environment for 1 week before the experiments. Rats were randomly divided into eight groups (*n* = 6) including sham-operated for 3 and 6 weeks, control (OA + the vehicle [water]) for 3 and 6 weeks, OA + morroniside (low, middle and high-dose) for 3 weeks, and OA + morroniside (low, middle and high-dose) for 6 weeks. The right knee joint of each rat was the experimental joint. The rat experimental model of OA was induced with anterior cruciate ligament transection in combination with resection of medial menisci (ACLT + MMx) as previously described 4,21. From the 4th week post-surgery (Fig. S1B), different doses of morroniside and the vehicle (water) were injected into the knee joints, and rats were not killed until the 3 or 6 weeks after injection, respectively.

### Histopathological scores

Semi-quantitative histopathological grading was performed according to a modified Mankin scoring system established for grading OA changes 22–24 Mankin scores normally consider five characteristics: structure, chondrocyte number, chondrocyte clustering, proteoglycan content and subchondral bone plate and/or tidemark change 25. Three sequential 3–5 μm sections (100 μm apart) of each sample were scored by two different blinded observers, for a maximum possible score of 15. Low scores are consistent with minor degenerative cartilaginous lesions, whereas high total scores are indicative of more pronounced cartilaginous changes.

Haematoxylin and eosin staining showed the morphology of chondrocytes, and the level of proteoglycans in the extracellular matrix was evaluated by Safarinin-O staining as previously described 4.

### Immunohistochemistry technique

The tibia articular sections of paraffin-embedded knees were incubated overnight at 4°C with primary antibody: p-AKT (1:100 dilutions) and p-ERK (1:100) subsequently, with secondary antibody (1:400) for 60 min. as described in the manufacturer’s instructions (Maixin Bio, Fuzhou, China). Diaminobenzidine was used to visualize the immunohistochemical reaction followed by being counterstained with haematoxylin. Finally, dark brown cells were considered to be positive. Photomicrographs were taken with OLYMPUS BX41 microscope equipped with a digital camera. The relative density of immunostaining (density/area) was measured using Image-Pro Plus 6.0 Software 4,10.

### TUNEL assay

*In situ* visualization of apoptotic cells were performed with TUNEL (terminal dexynucleotidyl transferase-mediated dUTP nick end labelling, TUNEL) assay according to the manufacturer’s instruction with In Situ Cell Death Detection Kit, POD (Roche Diagnostics, Shanghai, China), as previously described 10.

### Statistical analysis

All data were expressed as the mean ± SEM for three or five independent experiments for each group. The differences between the groups were examined for statistical significance using Student’s *t*-test and one-way anova with SPSS software (Shanghai, China). A value of *P* < 0.05 was considered as being significant.

## Results

### Effect of morroniside on human OA chondrocytes

Cultured human OA chondrocytes were treated with different doses of morroniside (0.1, 20 and 100 μM) for 24 hrs. The results of the MTT assay showed that 100 μM morroniside significantly enhanced the cell viability of human OA chondrocytes when compared with the untreated cells (Fig.1B, *P < 0.05), while 0.1 and 20 μM morroniside had no obvious effect on the cell viability of human OA chondrocytes (Fig.1B). Similarly, 100 μM morroniside significantly enhanced PCNA (a marker of cell proliferation) expression, as observed from western blotting analysis (Fig.1C, *P < 0.05). As the loss of COL2 and AGG are the primary event leading to the matrix depletion of cartilage, the levels of COL2 and AGG were detected with western blotting analysis. The levels of COL2 expression were enhanced in 0.1 and 20 μM morroniside-treated cells, while the levels of COL2 expression did not change in 100 μM morroniside-treated cells (Fig.1D, *P < 0.05). Aggrecan expression increased in a concentration-dependent manner of morroniside (Fig.1E, *P < 0.05, **P < 0.01). These results clearly show that morroniside can promote cell survival and matrix synthesis in human OA chondrocytes.

### Morroniside activates AKT and ERK in human OA chondrocytes

**Figure 2:**
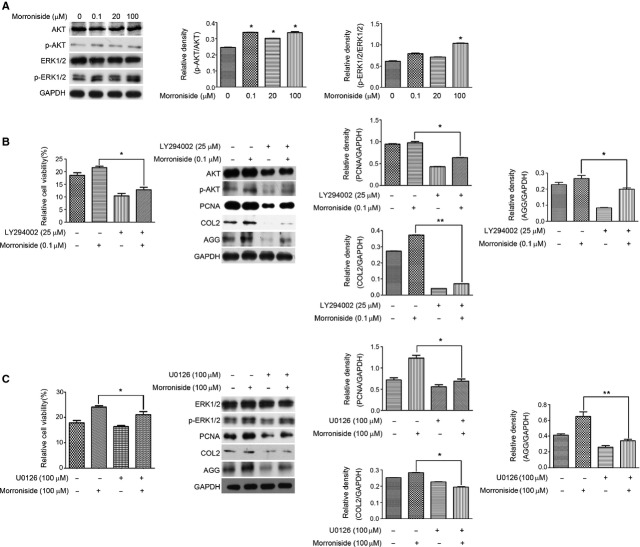
Morroniside activates AKT and ERK in human OA chondrocytes. (A) Cells were treated with different dose of morroniside (0.1, 20 and 100 μM) for 24 hrs. The levels of AKT, ERK, p-AKT and p-ERK were detected by western blotting analysis. (B) Cells were pre-treated with LY294002 (25 μM) for 2 hrs prior to treatment with morroniside (0.1 μM) for 24 hrs. The cell viability was measured by the MTT assay, and the levels of AKT, p-AKT, PCNA, COL2 and AGG were detected by western blotting analysis, respectively. (C) Cells were pre-treated with U0126 (100 μM) for 2 hrs prior to treatment with morroniside (100 μM) for 24 hrs. The cell viability was measured by the MTT assay, and the levels of ERK, p-ERK, PCNA, COL2 and AGG were detected by Western blotting analysis, respectively. The values represent the mean ± SEM of three to five independent experiments, each yielding similar results (*P < 0.05, **P < 0.01).

Compared with untreated cells, the levels of total AKT and ERK1/2 expression in morroniside-treated cells were not changed. However, three doses of morroniside (0.1, 20 and 100 μM) significantly up-regulated the level of p-AKT. Only 100 μM morroniside was sufficient to enhance the level of p-ERK1/2 (Fig.2A, *P < 0.05). Furthermore, the addition of LY294002 (25 μM) for 2 hrs dramatically attenuated the morroniside (0.1 μM)-stimulated increase in the cell viability, p-AKT, PCNA, COL2 and AGG level in human OA chondrocytes (Fig.2B, *P < 0.05, **P < 0.01, *versus* those treated only with morroniside). Similarly, the addition of U0126 (100 μM) for 2 hrs also attenuated the morroniside (100 μM)-stimulated increase in the cell viability, p-ERK, PCNA, COL2 and AGG level in human OA chondrocytes (Fig.2C, *P < 0.05, **P < 0.01, *versus* those treated only with morroniside). Comparison of the effect of morroniside on p-AKT and p-ERK revealed that they were not synchronized and that p-AKT is more sensitive than p-ERK to morroniside treatment in human OA chondrocytes. Moreover, the inhibitory effect of LY294002 on COL2 expression was stronger than that of U0126 (Fig.2B and C). AKT and ERK activation thus can contribute to the morroniside-stimulated promoting effect on cell viability and matrix synthesis in human OA chcondrocytes.

**Figure 3:**
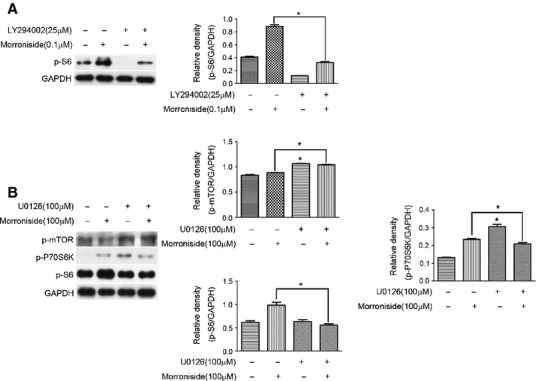
The role of S6, P70S6K and mTOR in the regulatory mechanism of AKT and ERK in morroniside-treated OA chondrocytes. (A) Cells were pre-treated with LY294002 (25 μM) for 2 hrs prior to treatment with morroniside (0.1 μM) for 24 hrs. The level of p-S6 was then detected by western blotting analysis. (B) Cells were pre-treated with U0126 (100 μM) for 2 hrs prior to treatment with morroniside (100 μM) for 24 hrs, and the levels of p-P70S6K and p-S6 were detected by western blotting analysis. The values represent the mean ± SEM of three to five independent experiments, each yielding similar results (*P < 0.05).

To explore the regulatory mechanism of activated AKT and ERK in morroniside-treated human OA chondrocytes, the mTOR/P70S6K/S6 pathway that is the main pathway to regulate protein synthesis, was investigated. The result of Figure3A showed that morroniside only enhanced the level of p-S6, which was blocked dramatically by Ly294002 (Fig.3A, *P < 0.05, *versus* those treated only with morroniside). Simultaneously, 100 μM morroniside enhanced the levels of p-P70S6K and p-S6, but not p-mTOR. The addition of U0126 attenuated the morroniside-stimulated increase in p-P70S6K (*P* = 0.0038) and p-S6 level in human OA chondrocytes (Fig.3B, *P < 0.05, *versus* those treated with only morroniside). However, U0126 caused a significant increase in p-mTOR and p-P70S6K level (Fig.3B, *P < 0.05, *versus* the untreated group). Therefore, the regulatory mechanism of AKT and ERK may be associated with the phosphorylation of S6, P70S6K, or mTOR in morroniside-treated OA chondrocytes.

### Morroniside ameliorates the cartilage damage in a rat experimental model of OA with the increase of p-AKT and p-ERK

**Figure 4:**
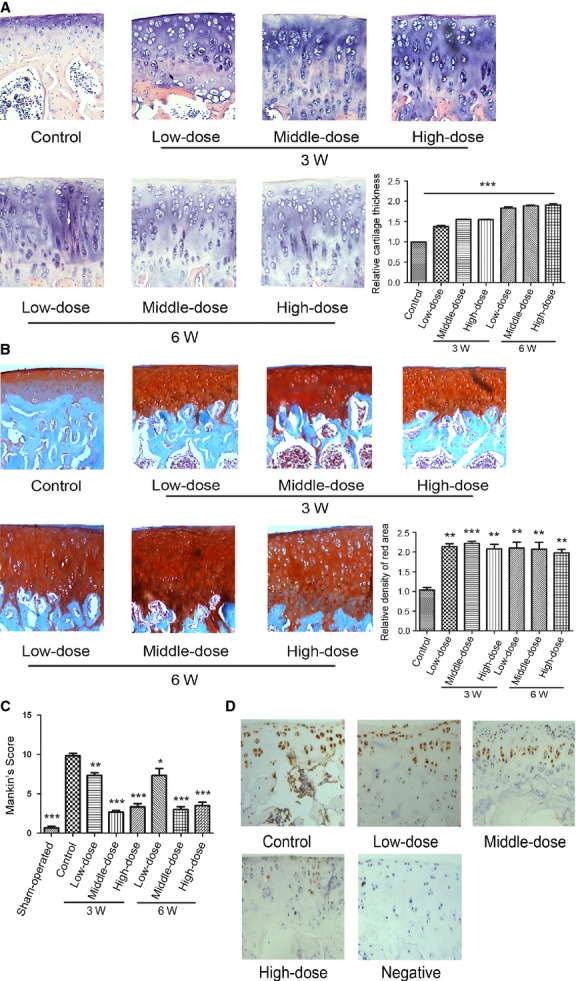
Morroniside ameliorates the cartilage damage in a rat experimental model of OA Specimens were longitudinally cut into 3–5 μm sections. (A) Sections in different treated groups were examined using haematoxylin and eosin staining (original magnification ×100). (B) Sections in different treated groups were examined using Safranin O-fast green staining (original magnification ×100). The index of matrix production was evaluated by relative density of red area. The values represent the mean ± SEM of three to five independent experiments, each yielding similar results (**P < 0.01, ***P < 0.001). (C) Histopathological scores were performed by Mankin’s score. (D) Sections in morroniside-treated 6-week group were performed with TUNEL assay (original magnification ×100).

According to the data in human OA chondorcytes, the three doses morroniside chosen, including 1 × 10^−5^ g/kg (equal to the dose to activate AKT in culture cells, low-dose), 1 × 10^−2^ g/kg (equal to the dose to activate ERK in culture cells, high-dose) and 5 × 10^−4^ g/kg (middle-dose), were injected into the knee joints, and rats were not killed until the 3 or 6 weeks after injection, respectively. The intra-articular injection with different doses of morroniside all led to the increase of cartilage thickness significantly (Fig.4A, ***P < 0.001, *versus* control group). Specifically, the increase of cartilage thickness in morroniside-treated 6 week group was more than that in morroniside-treated 3 week group (Fig.4A,*P < 0.05). Concomitantly, the density of proteoglycans in cartilage matrix from morroniside-treated group was more than control group (Fig.4B, *P < 0.05, **P < 0.01, ***P < 0.001, *versus* control group). The severity of cartilage destruction was then scaled using Mankin’s score 4. The score of control group (9.83 ± 0.752) was much higher than that of the sham-operated group (0.67 ± 0.519), indicating the reliability of our OA model. The scores of morroniside-treated 3 week group (low-dose [7.33 ± 0.574], middle-dose [2.67 ± 0.516] and high-dose [3.33 ± 1.032]) and 6 week group (low-dose [7.0 ± 1.526], middle-dose [3.0 ± 0.89] and high-dose [3.5 ± 0.806]) were lower than that of control groups (9.83 ± 0.752; Fig.4C, *P < 0.05, **P < 0.01, ***P < 0.001, *versus* control group). Meanwhile, the results of TUNEL assay showed that the apoptotic chondrocytes in morroniside-treated 6 week group were fewer than that in control group (Fig.4D). These data indicate that different doses of morroniside can ameliorate the cartilage damage with the decrease of apoptotic chondrocytes in a rat experimental model of OA.

**Figure 5:**
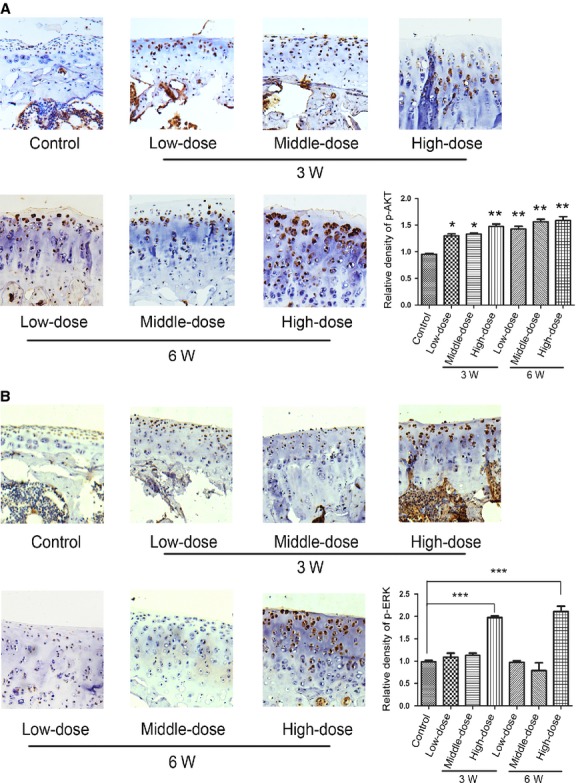
Morroniside enhanced the levels of p-AKT and p-ERK expression in a rat experimental model of OA. Specimens were longitudinally cut into 3–5 μm sections and the levels of p-AKT (A) and p-ERK (B) expression were detected by immunohistochemisty technique (original magnification ×100). The index of protein expression was evaluated by the fold change of the relative density/area. The values represent the mean ± SEM of three to five independent experiments, each yielding similar results (*P < 0.05, **P < 0.01, ***P < 0.001).

Additionally, the levels of p-AKT or p-ERK in superficial and middle layer of cartilage increased significantly in morroniside-treated groups (Fig.5A and B,*P < 0.05, **P < 0.01, ***P < 0.001, *versus* control group). Therefore, intra-articular injection with definite doses of morroniside, in part, ameliorated the cartilage damage with the increase of p-AKT and p-ERK levels.

## Discussion

Here, we demonstrated that morroniside promoted cell survival and matrix synthesis in cultured human OA chondrocytes. The activation of AKT and ERK contributed to the effect of morroniside. Furthermore, the intra-articular injection of morroniside can ameliorate the cartilage damage in a rat experimental model of OA. These findings suggest that morroniside has chondroprotective effect on OA chondrocytes, and may have the therapeutic potential for OA treatment.

It is well-known that inhibiting apoptosis/maintaining survival of chondrocytes is a good approach for OA therapy 1,2,10. Here, the treatment of morroniside promoted cell survival in human OA chondrocytes or protected cells against apoptosis a rat experimental model of OA, similar to the effects of morroniside on nerve cells and human embryonic lung fibroblast cells 7,8. Furthermore, the levels of COL2 and AGG can be enhanced in morroniside-treated human OA chondrocytes, suggesting the promoting effect of morroniside on matrix synthesis. Combined with the data that morroniside ameliorated articular cartilage destruction in a rat experimental model of OA, it is confirmed that morroniside has exhibited chondroprotective effect on OA chondrocytes.

AKT and its downstream effectors are responsible for propagating signals from many extracellular stimuli, such as berberine, insulin-like growth factor (IGF)-1 and nicotine, to exert their chemopreventive effects in chondrocytes 4,13,20,26,27. For example, berberine has promoted cell proliferation of IL-1β–stimulated chondrocytes *via* activated AKT 4. The PI3K/AKT pathway primarily has transduced the IGF-1 effect on COL2 expression in rat endplate chondrocytes stimulated by IGF-I 27. Thus, AKT signalling appears to be one of the targets of these extracellular factors for exerting their chondroprotective effects in chondrocytes. Here, the promoting effect of morroniside on cell viability and the levels of PCNA, COL2 and AGG expression in OA chondrocytes can be blocked by the inhibitor of PI3K/AKT, implying that AKT may be one of targets of morroniside for exerting its chondroprotective effects in human OA chondrocytes. We further observed that only S6, one of the 40S ribosomal subunits that promote protein synthesis, rather than mTOR and P70S6K, was the downstream of activated AKT in morroniside-treated human OA chondrocytes. This finding is consistent with other authors’ studies 28,29 and our previous study in nicotine-stimulated chondrocytes 20. Because S6 is known to possess an RxRxxS/T motif, which can be phosphorylated by AGC kinase family members such as AKT, RSK and p70S6K, PI3K/AKT-mediated phosphorylation of S6 can be accomplished through an mTOR-independent manner in human OA chondrocytes. Therefore, it is suggested that the AKT/S6 pathway triggered by morroniside is involved in the regulation of morroniside on cell survival and matrix synthesis in human OA chondrocytes.

Although ERK are known to play a certain role in OA pathogenesis, the role of activated ERK is not always consistent. Vorinostat exhibits anti-osteoarthritic activities through the inhibition of iNOS and MMP expression, p38 and ERK phosphorylation 30. However, the activation of the ERK1/2 signalling pathway by raloxifene can prevent caspase-3-dependent apoptosis induced by tumour necrosis factor-α in human chondrocytes by acting as an anti-apoptotic signalling event 16. The inconsistent effect of ERK activation on chondrocytes may be due to the type of stimuli or the context of cells. Our data that the MEK/ERK inhibitor attenuated the promoting effect of morroniside on cell survival and matrix synthesis supported the view that activated ERK positively regulated cell survival of OA chondrocytes, serving as one of targets of morroniside as AKT did.

Several lines of evidence have shown that P70S6K is involved in the regulatory mechanisms of ERK 31,32. FGFs can activate p70S6K by means of the ERK pathways in chondrogenic and gliogenic specification of mouse mesencephalic neural crest cells 32. Moreover, the phosphorylation of P70S6K does not always depend on mTOR activation. Osteopontin-induced phosphorylation of p70S6K at Thr-389 does not depend on mTOR, and the phosphorylation of p70S6K at Thr-421/Ser-424 is controlled by the MEK/ERK pathway in breast cancer cells 33. Our data that ERK inhibition attenuated the promoting effect of morroniside on p-P70S6K and p-S6 rather than p-mTOR, also indicated that the phosphorylation of P70SK at Thr-389 and S6 at Ser235/236 was, at least partly, independent of p-mTOR. The ERK/P70S6K/S6 pathway may contribute to the regulation of morroniside in human OA chondrocytes. On the other hand, ERK signalling has been reported to regulate mTOR signalling in previous studies 34,35. For example, polycystin-1 can control the mTOR pathway by inhibiting ERK-mediated phosphorylation of TSC 35. Consistent with them, in the present study, ERK inhibition also regulated the phosphorylation of mTOR and P70S6K, while activated ERK may negatively regulate the mTOR or P70S6K-related pathway in morroniside-treated human OA chondorocytes. Further research into the regulatory mechanism of ERK on mTOR signalling should be explored.

Previous studies have reported the differential regulation of AKT and ERK 36,37. For example, oxidative stress inhibits IGF-I induction of chondrocyte proteoglycan synthesis through differential regulation of PI3K/AKT and MEK/ERK signalling pathways 36.

In the present study, p-AKT was more sensitive than p-ERK to morroniside treatment and the effect of activated AKT on COL2 expression was stronger than that of ERK in morroniside-treated chondrocytes. We speculate that the regulation of activated AKT on collagen synthesis predominated over that of ERK, and activated AKT is inclined to regulate collagen synthesis in morroniside-treated OA chondrocytes. Further study would be necessary to probe the mechanism of AKT and ERK in OA pathogenesis.

**Figure 6:**
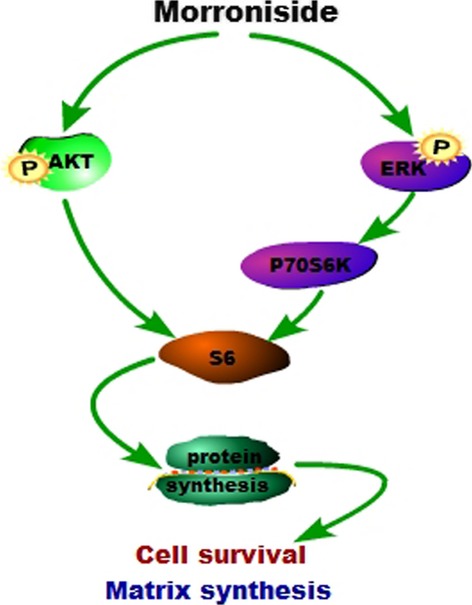
The tentative mechanism of the chondroprotective effect of morroniside on OA chondrocytes.

In conclusion, morroniside can exert its chondroprotective effect on OA chondrocytes in human OA chondrocytes and a rat OA model. The AKT and ERK activation can contribute to the regulation of morroniside in OA chondrocytes (Fig.6).

## Supporting Information

**Figure S1** Articular cartilage surface of human knee joint and rat knee joint.
