# Supplementary material for: Protein kinase B and extracellular signal-regulated kinase contribute to the chondroprotective effect of morroniside on osteoarthritis chondrocytes
Source: J Cell Mol Med. 2015 Mar 5;19(8):1877–86. doi: 10.1111/jcmm.12559 (PMC4549038; doi:10.1111/jcmm.12559)
Supplement: Supplementary file 1 [file jcmm0019-1877-sd1.zip › figure legend of supple.1.docx]

**Supplementary Fig. 1 Articular cartilage surface of human knee joint and rat knee joint.** (A) Articular cartilage from patients was dissected from the femoral condyle and tibial plateau (the section of obtained cartilage indicated by black arrows). (B) Articular cartilage from the 4th week post-surgery rats (the section of obtained cartilage indicated by black arrows).
