# Supplementary figures and images for: Protein kinase B and extracellular signal-regulated kinase contribute to the chondroprotective effect of morroniside on osteoarthritis chondrocytes
Source: J Cell Mol Med. 2015 Mar 5;19(8):1877–86. doi: 10.1111/jcmm.12559 (PMC4549038; doi:10.1111/jcmm.12559)

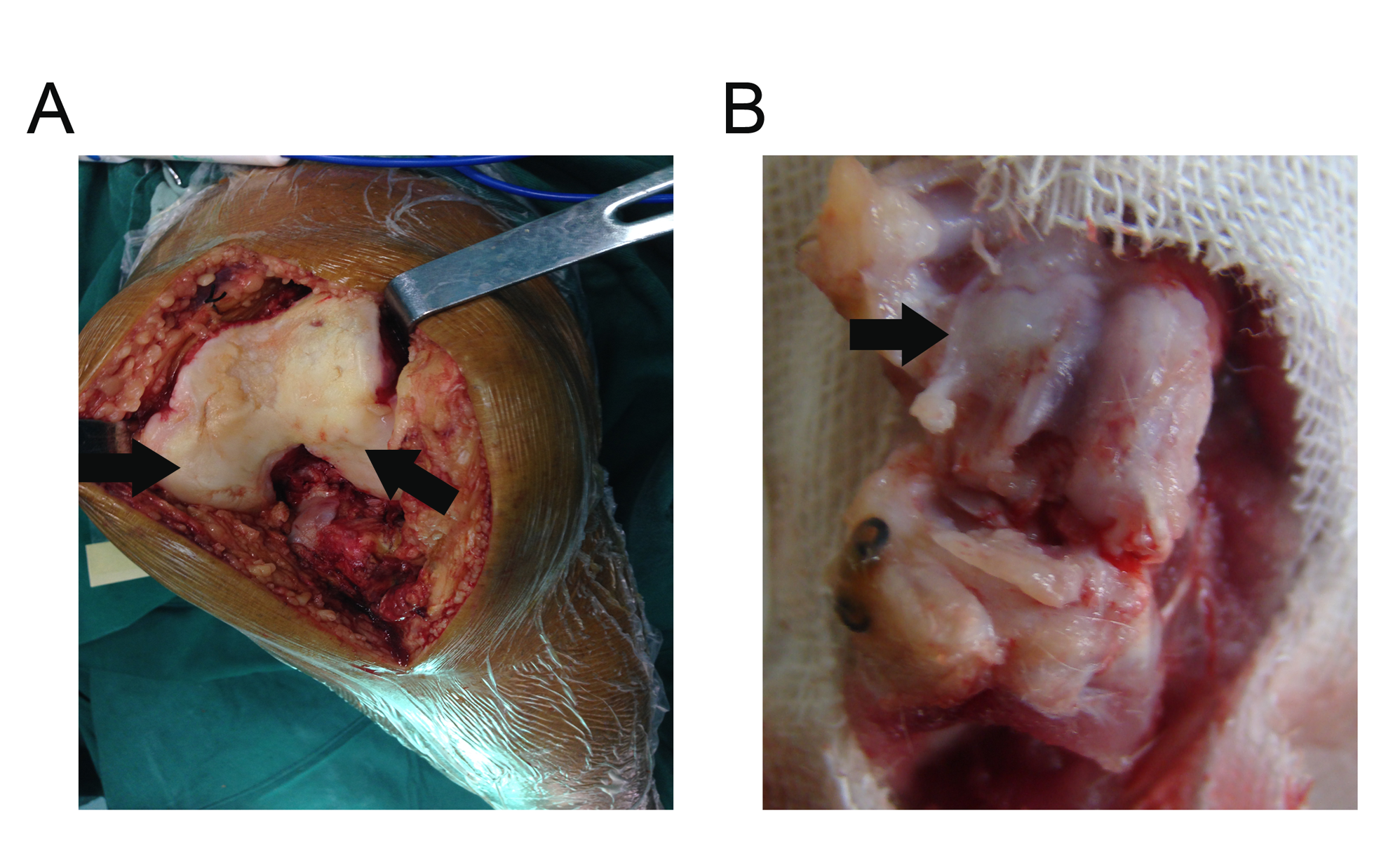

Supplement: Supplementary file 1 [file jcmm0019-1877-sd1.zip › supple 1.tif]
